# Three dimensional Ni_3_S_2_ nanorod arrays as multifunctional electrodes for electrochemical energy storage and conversion applications†

**DOI:** 10.1039/c9na00633h

**Published:** 2019-11-22

**Authors:** Kexin Cui, Jincheng Fan, Songyang Li, Moukaila Fatiya Khadidja, Jianghong Wu, Mingyu Wang, Jianxin Lai, Hongguang Jin, Wenbin Luo, Zisheng Chao

**Affiliations:** College of Materials Science and Engineering, Changsha University of Science and Technology Changsha Hunan 410114 China fanjincheng2009@163.com chao_zs@aliyun.com; College of Health Science and Environmental Engineering, Shenzhen Technology University Shenzhen Guangdong 518118 China

## Abstract

The increasing demand for energy and environmental protection has stimulated intensive interest in fundamental research and practical applications. Nickel dichalcogenides (Ni_3_S_2_, NiS, Ni_3_Se_2_, NiSe, *etc.*) are promising materials for high-performance electrochemical energy storage and conversion applications. Herein, 3D Ni_3_S_2_ nanorod arrays are fabricated on Ni foam by a facile solvothermal route. The optimized Ni_3_S_2_/Ni foam electrode displays an areal capacity of 1602 µA h cm^−2^ at 5 mA cm^−2^, excellent rate capability and cycling stability. Besides, 3D Ni_3_S_2_ nanorod arrays as electrode materials exhibit outstanding performances for the overall water splitting reaction. In particular, the 3D Ni_3_S_2_ nanorod array electrode is shown to be a high-performance water electrolyzer with a cell voltage of 1.63 V at a current density of 10 mA cm^−2^ for overall water splitting. Therefore, the results demonstrate a promising multifunctional 3D electrode material for electrochemical energy storage and conversion applications.

## Abstract 2

3D Ni_3_S_2_ nanorod arrays/Ni foam as multifunctional electrodes for electrochemical energy storage and conversion applications have been achieved, demonstrating outstanding galvanization charging/discharging, hydrogen evolution reaction and oxygen evolution reaction performances.

## Graphical abstract

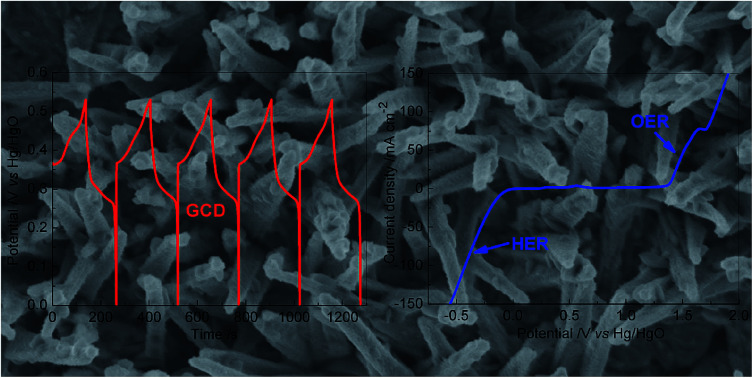

## 1. Introduction

The increasing worldwide application of electronic devices has put forward great challenges for clean and safe energy conversion and storage systems.^1–5^ Various energy conversion and storage devices have been designed and fabricated, such as supercapacitors,^6–9^ and alkali metal (Li, Na and K)-ion,^10–21^ divalent Mg-ion^22,23^ and multivalent Al-ion batteries.^24–26^ Among them, supercapacitors, also called electrochemical capacitors, have attracted increasing attention due to their high power densities, long cycle life, and fast recharge.^27–32^ Besides, as an effective route of energy conversion and storage, the hydrogen (H_2_) evolution reaction for water splitting with catalyst materials has been explored.^33–37^ Generally, electrode materials are considered to play significant roles in determining the performances of the energy conversion and storage systems.^38–41^

In recent years, Ni_3_S_2_, a typical nickel sulfide with a high theoretical capacity of 2412 F g^−1^, has been reported to demonstrate outstanding electrochemical performances for supercapacitors and H_2_ evolution due to its low cost, good conductivity, higher electrochemical activities and environmental friendliness.^42–44^ Li *et al.* synthesized Ni_3_S_2_ nanoparticles by simple mechanical alloying as the electrode for a supercapacitor.^45^ The supercapacitor demonstrated a specific capacitance of 911 F g^−1^ at 0.5 A g^−1^. However, there is significant gap between its actual and theoretical capacitance. To improve the performances of Ni_3_S_2_-based supercapacitors, various Ni_3_S_2_-composites are designed and fabricated as the electrode materials.^47–50^ Zhou *et al.* reported Ni_3_S_2_ nanorod@Ni(OH)_2_ nanosheet core–shell nanostructures on a three dimensional (3D) graphene network as the electrode for a supercapacitor, which exhibited a large specific capacitance of 1037.5 F g^−1^ at 5.1 A g^−1^ and a good cycling stability.^46^ Wang *et al.* designed and synthesized a multiple electrode structure based on nano Ni_3_S_2_ and carbon nanotubes.^50^ It exhibited a remarkable electrochemical performance with an areal specific capacitance of 13 400 mF cm^−2^ at a current density of 10 mA cm^−2^. In addition, Ni_3_S_2_ is also one of the important catalyst materials for H_2_ evolution from water splitting.^51–54^ For instance, Zhang *et al.* synthesized a mesoporous Ni_3_S_2_ particle electrocatalyst on Ni foam by a hydrothermal method, which demonstrated excellent catalytic activity and rapid reaction kinetics.^54^ The optimized Ni_3_S_2_ electrocatalyst exhibited ultralow overpotentials of 213 mV at 10 mA cm^−2^ with a very low Tafel slope of 45 mV dec^−1^ in alkaline media.

Here, we synthesized a three dimensional Ni_3_S_2_ nanorod array electrode on nickel foam (Ni_3_S_2_/NF) by a facile solvothermal route. The characterization and electrochemical measurements for the Ni_3_S_2_ nanorod array electrode were investigated, systematically, to estimate its potential ability in electrochemical energy storage and conversion applications. The optimized Ni_3_S_2_/NF displays outstanding areal capacity (1602 µF cm^−2^ at 5 mA cm^−2^), excellent rate capability (64% rate retention in the current density ranges of 2.5 to 25 mA cm^−2^) and cycling stability (86.94% retention after 3400 cycles at 15 mA cm^−2^). Further, the Ni_3_S_2_/NF catalyst also exhibits superior performances in the hydrogen evolution reaction (HER) and oxygen evolution reaction (OER). In particular, the 3D Ni_3_S_2_ nanorod array electrode is shown to be a high-performance water electrolyzer with a cell voltage of 1.63 V at a current density of 10 mA cm^−2^ for overall water splitting. Therefore, Ni_3_S_2_/NF multifunctional nanostructures can be extensively used for broad application prospects in electrochemical energy conversion and storage systems.

## 2. Experimental section

### 2.1. Fabrication of 3D Ni_3_S_2_ nanorod arrays

The Ni_3_S_2_ nanorod arrays were grown on the Ni foam substrate using a facile solvothermal method. Prior to the fabrication of Ni_3_S_2_ nanorod arrays, Ni foam with a size of 1 cm × 2 cm was ultrasonically cleaned in acetone, 3 M HCl, ethanol and deionized water in sequence to remove the possible residual inorganic/organics on the surface. 2.13 g of Na_2_S·9H_2_O were added into 20 ml methanol and ultrasonically dispersed for 20 min to obtain a homogeneous solution. Subsequently, the solution was transferred to a Teflon-lined stainless steel autoclave (25 ml). Finally, a piece of pre-treated Ni foam was carefully immersed in the solution. The autoclave was sealed and kept at 120 °C for 16 h. After the reaction, the autoclave was cooled down to room temperature, and the Ni foam was carefully taken out from the reaction vessel, then rinsed in deionized (DI) water to remove any residual salt, and dried in air. The sample was marked as S-120-16. Similarly, another three samples were prepared at 100 °C for 16 h (S-100-16), 140 °C for 16 h (S-140-16) and 120 °C for 18 h (S-120-18), respectively.

### 2.2. Materials characterization

The crystallographic information about Ni_3_S_2_ on Ni foam was obtained by X-ray diffraction (XRD). The XRD patterns with diffraction intensity *versus* 2*θ* were recorded on a Bruker D8 using Cu Kα radiation (*λ* = 0.15405 nm). Scanning electron microscopy (SEM) images were recorded using an FIT Nano430. FEI Tecnai G2 transmission electron microscopy (TEM) was performed for Ni_3_S_2_ on Ni foam. X-ray photoelectron spectroscopy (XPS) was carried out on a VG Escalab 210 spectrometer fitted with an Al Kα X-ray source. Raman spectroscopy of the as-grown sample was performed using a Raman spectrometer (Horiba JY LabRAM) with an argon ion laser (*λ* = 514 nm).

### 2.3. Electrochemical measurements

Electrochemical measurements (cyclic voltammetry and electrochemical impedance spectroscopy) were performed in a traditional three-electrode cell using a CHI 660E electrochemical workstation. The as-synthesized sample on Ni foam (1 cm × 2 cm) was directly used as the working electrode. Platinum foil (Pt, 1 cm × 1 cm) served as the counter electrode and the Hg/HgO electrode acted as the reference electrode. Cyclic voltammetry (CV), electrochemical impedance spectroscopy (EIS), galvanization charge/discharge (GCD) and cycling performance measurements were carried out in 6 M aqueous KOH. The areal capacity can be calculated using the equation *C*_A_ = *I* × Δ*t*/(Δ*V* × *S*), here, *I* (A) is the galvanostatic current applied to the electrode, Δ*t* (s) is the discharge time, Δ*V* (V) is the potential window, and *S* (cm^−2^) is the geometric surface area of the working electrode. Prior to the electrochemical test, the samples were immersed in 150 ml 6 M KOH electrolyte solution for 3 h.

The HER and OER tests were performed in 1 M KOH solution. The sample (1 cm × 2 cm), platinum foil (1 cm × 1 cm) and a Hg/HgO (1.0 M KOH) electrode were the working, counter and reference electrodes, respectively. All the potentials used in the study were converted to potentials *versus* the reversible hydrogen electrode (RHE) using the following equation: *E*_RHE_ = *E*_Hg/HgO_ + 0.059pH + 0.14 V. The overpotential was calculated using the equation *η* = *E*_RHE_ − 1.23 V; the Tafel slope was obtained from the Tafel equation *η* = *b* log *j* + *a*, where *a* is a constant, *j* is the current density and *b* is the Tafel slope. In order to get closer to the actual situation, here, the potential value of each electrode was not corrected by compensating the *iR* drop.

## 3. Results and discussion

**Fig. 1:**
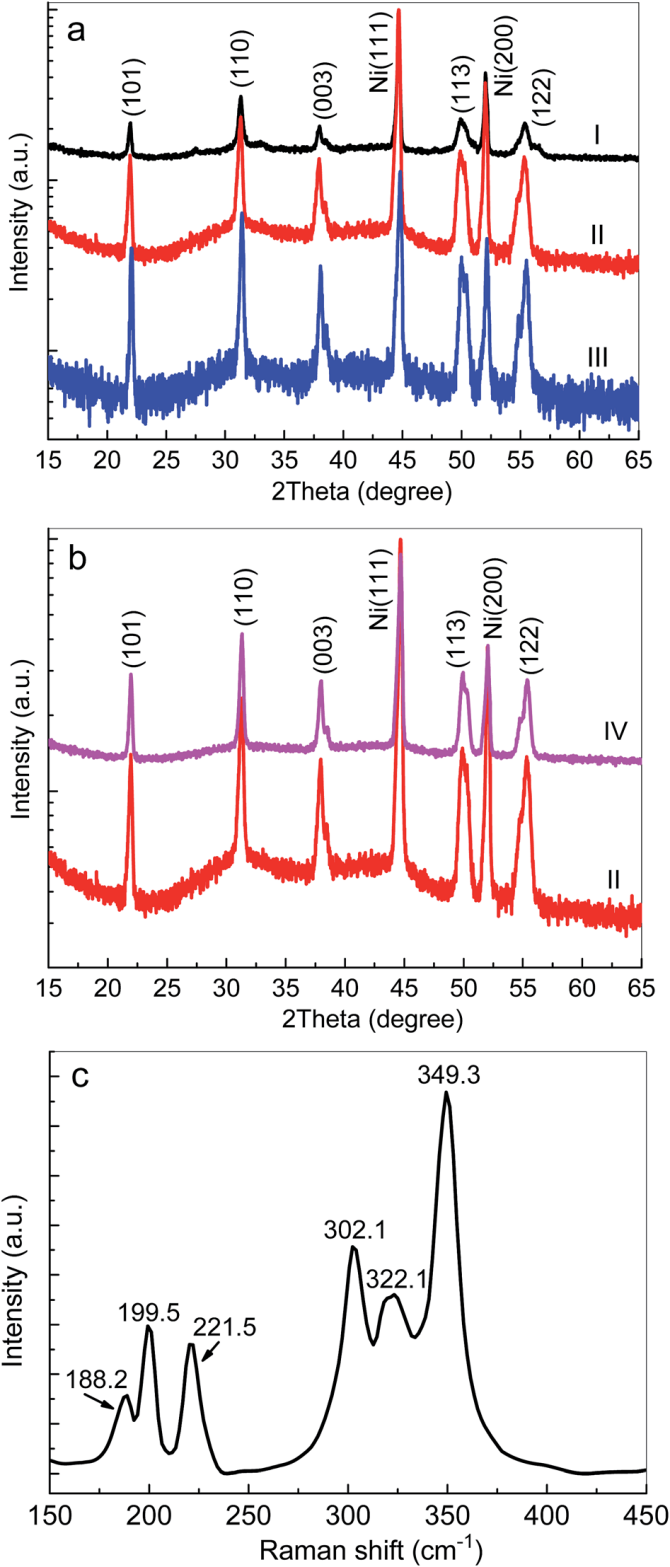
(a and b) XRD patterns of Ni_3_S_2_ nanorods fabricated on Ni foam substrates: (I) 100 °C, 16 h; (II) 120 °C, 16 h; (III) 140 °C, 16 h; (IV) 120 °C, 18 h. (c) The typical Raman spectrum of the Ni_3_S_2_ nanorods on Ni foam (S-120-16).

Fig. 1a and b show XRD patterns of Ni_3_S_2_ fabricated on Ni foam substrates. Two sharp peaks around 44.6° and 52.1° were attributed to the Ni foam substrate (JCPDS 04-0805). The peaks at about 21.9°, 31.3°, 37.9°, 49.8° and 55.3° were detected and were assigned to the (101), (110), (003), (113), and (122) planes of hexagonal Ni_3_S_2_ (JCPDS no. 44-1418), respectively. The absence of other peaks indicated the purity of the synthesized material.

To further study the crystal structure of the as-prepared samples, the Raman spectra of Ni_3_S_2_/Ni foam were characterized, as shown in Fig. 1c. Obviously, the Raman spectra of Ni_3_S_2_ prepared on Ni foam demonstrated various peaks at ∼188.2 cm^−1^, ∼199.5 cm^−1^, ∼221.5 cm^−1^, ∼302.1 cm^−1^, ∼322.1 cm^−1^ and ∼349.3 cm^−1^, which can be attributed to the vibration of Ni_3_S_2_.^55^ No other impurity peaks, such as Ni(OH) or NiO, in the Raman spectra were observed, which shows that Ni_3_S_2_ on Ni foam with high purity had been successfully fabricated.

**Fig. 2:**
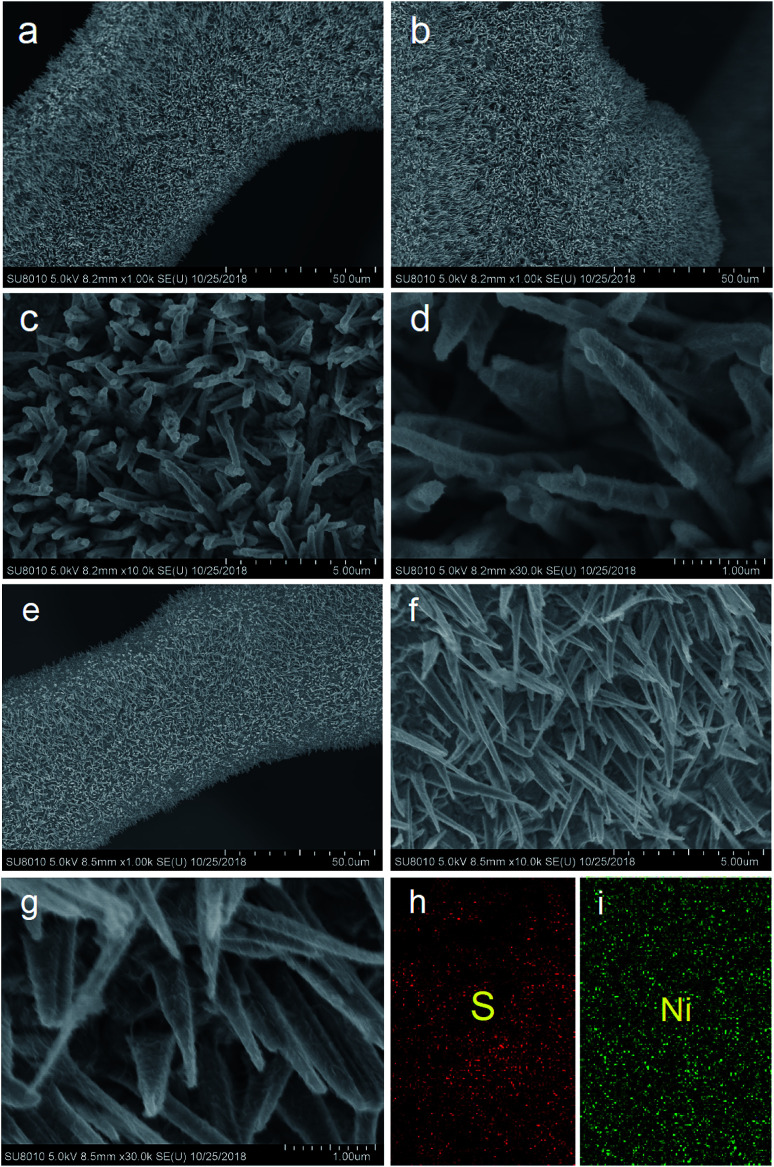
SEM and EDS images of the Ni_3_S_2_ nanorods on Ni foam with different magnifications, (a) and (b) ×1k; (c) ×10k; (d) ×30k; (e) and (f) ×1k; (g) ×10k; (h) EDS image of the S element; (i) EDS image of the Ni element. (a)–(d) images obtained for S-120-16; (e)–(i) images obtained for S-120-18.

The SEM images of the Ni_3_S_2_ samples are presented in Fig. 2 and S1.† It can be seen that large-scale oriented uniform Ni_3_S_2_ nanorods with a length of 2–4 µm were fabricated on Ni foam substrates. Ni_3_S_2_ nanorod arrays are standing closely to each other, and the channels between the Ni_3_S_2_ nanorods may provide facile pathways for electrolyte diffusion. The enlarged SEM images (Fig. 2c, d, f and g) show that Ni_3_S_2_ nanorods have a rough surface and some Ni_3_S_2_ nanoparticles were grown on the surface of nanorods for S-120-16. The EDS analysis is performed to investigate the composites of S-120-18 (Fig. 2h, i, and S2†). S and Ni element mappings are shown in Fig. 2h and i, indicating their uniform distributions in Ni_3_S_2_.

**Fig. 3:**
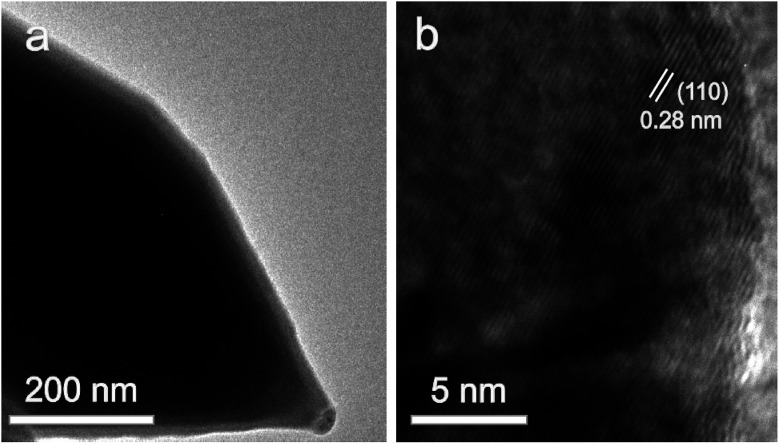
The typical TEM (a) and HRTEM (b) images of Ni_3_S_2_ nanorods.

Fig. 3 shows the typical TEM and high-resolution TEM (HRTEM) images of Ni_3_S_2_ nanorods (S-120-16). Obviously, the TEM image of Ni_3_S_2_ reveals the nanorod nature and the HRTEM image displays the clear lattice fringes with *d*-spacings of about 0.28 nm, which are attributed to the (110) planes of Ni_3_S_2_.

**Fig. 4:**
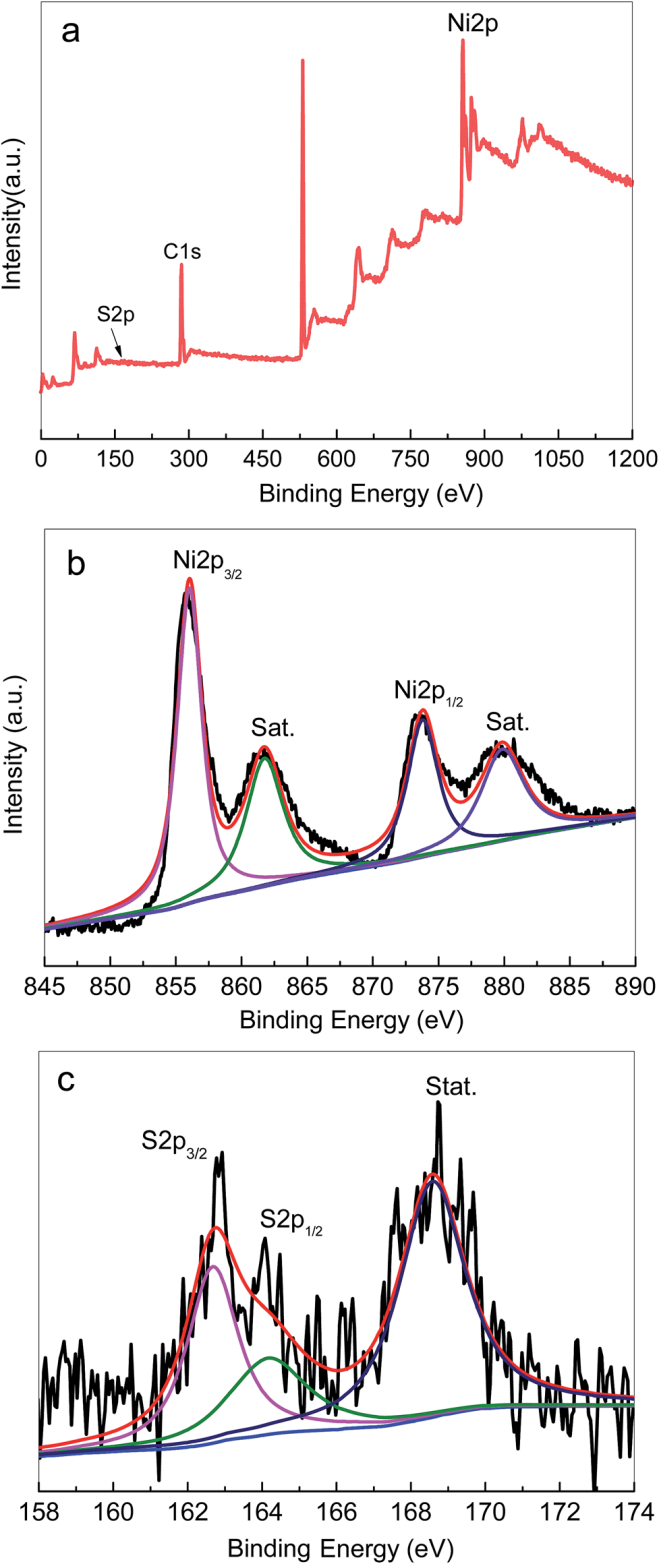
The typical XPS spectra of the Ni_3_S_2_ nanorods on Ni foam (S-120-16): (a) survey scan, (b) Ni 2p, (c) S 2p.

XPS spectra were recorded to further verify the composition and elemental valence states of the Ni_3_S_2_ samples. Fig. 4 displays the typical XPS spectra of the samples. The survey spectrum shows the presence of Ni and S elements (Fig. 4a), which was consistent with the EDS results. As shown in Fig. 4b, the high-resolution spectra of Ni 2p can be divided into two spin orbit doublets and two shakeup satellite peaks. The two main peaks centred at 855.8 eV and 873.4 eV are assigned to Ni 2p_3/2_ and Ni 2p_1/2_ accompanied by distinct satellite peaks, respectively, and the splitting between the two peaks is 17.6 eV. The obtained results indicated the coexistence of Ni^2+^ and Ni^3+^ in Ni_3_S_2_ nanorods.^56–59^Fig. 4c presents the high-resolution spectrum for the S 2p region. The two doublet peaks at 162.7 eV and 164.2 eV were detected, corresponding to S 2p_3/2_ and S 2p_1/2_. The XPS results are in good agreement with those of XRD and Raman spectra, confirming the successful fabrication of Ni_3_S_2_ on Ni foam by the solvothermal method.

### 3.1. Capacitive performance of the 3D Ni_3_S_2_ nanorod arrays

**Fig. 5:**
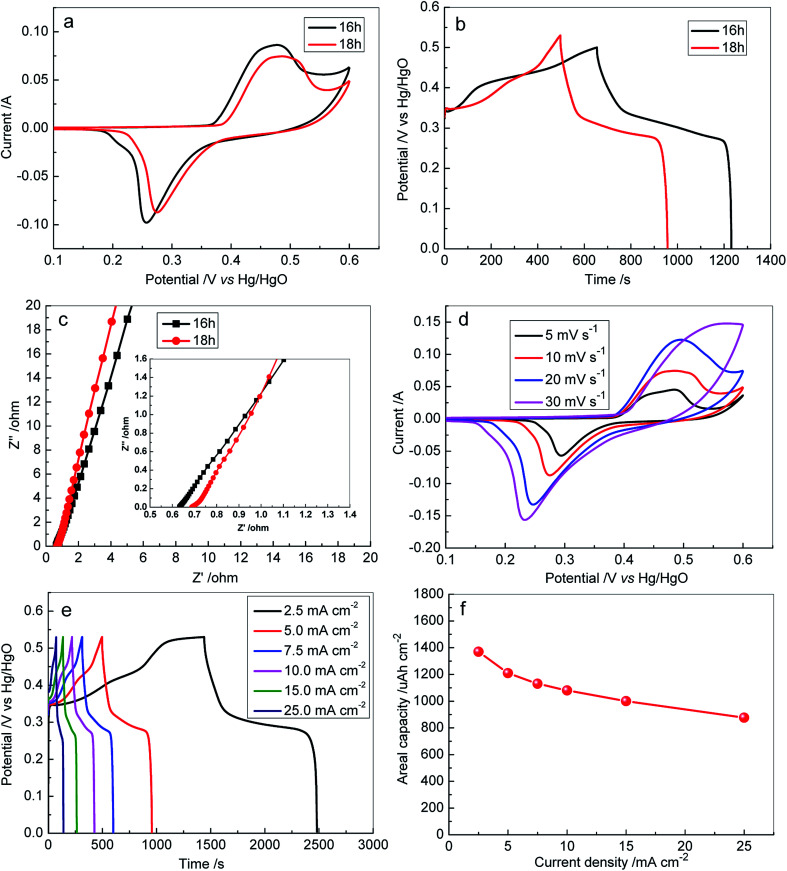
(a) CV curves of S-120-16 and S-120-18 electrodes at a scan rate of 10 mV s^−1^; (b) GCD profiles obtained for S-120-16 and S-120-18 electrodes at a current density of 5 mA cm^−2^; (c) Nyquist plots of S-120-16 and S-120-18 electrodes; (d) CV curves obtained for the S-120-18 electrode at different scan rates; (e) GCD profiles obtained for the S-120-18 electrode at different current densities; (f) the areal capacity calculated from the discharge process at different current densities.

The electrochemical performances of 3D Ni_3_S_2_ nanorod arrays were investigated by the three-electrode measurements with 6 M KOH as the electrolyte. Fig. 5a displays the CV curves for 3D Ni_3_S_2_ nanorod arrays prepared with different growth times at 120 °C (S-120-16 and S-120-18). It is observed that the samples possess a pair of redox peaks at ∼0.48 V (positive sweep) and ∼0.26 V (negative sweep), corresponding to the redox response of the reversible transition between Ni (+2) and Ni (+3), which implied that the battery-like behaviour of Ni_3_S_2_/NF samples and the energy storage of the material should come from the faradaic reaction.^60^ The reversible faradaic reaction could be described as the following equation:Ni_3_S_2_ + *x*OH^−^ ↔ Ni_3_S_2_(OH) + *x*e

The GCD of the samples was also measured at a current density of 5 mA cm^−2^, as shown in Fig. 5b. It can be seen that the samples exhibited long charge/discharge profiles, which are well matched with the CV results (Fig. 5a). The discharge duration for S-120-16 and S-120-18 was 577 s and 460 s, and their calculated areal capacities were 1511 µA h cm^−2^ and 1210 µA h cm^−2^ at 5 mA cm^−2^, respectively.

To explore the effect of internal resistance characteristics in promoting such an ideal performance of 3D Ni_3_S_2_ nanorod arrays, EIS tests were carried out and the obtained results are shown in Fig. 5c. From the fitted EIS circuit, the equivalent series resistance (*R*_s_) can be estimated, including the inherent resistance of electroactive materials, the bulk resistance of the electrolyte, and interfacial resistance at the active materials/current collector interface. It was found that S-120-16 and S-120-18 had similar *R*_s_ values, 0.65 Ω and 0.72 Ω, respectively, suggesting the high conductivity of Ni_3_S_2_ nanorods and the good electrical contact between Ni_3_S_2_ nanorods and Ni foam substrates.

To investigate the rate capacity of the 3D Ni_3_S_2_ nanorod array electrode, CV was conducted at different scan rates. Fig. 5d presents the CV curves at the scan rates of 5, 10, 20, and 30 mV s^−1^ for S-120-18. Obviously, the characteristic redox peak in each voltammogram increases with respect to the scan rates. Furthermore, the shape of the CV curves was highly stable, indicating that the 3D Ni_3_S_2_ nanorod array electrode had a stable electrochemical performance. To evaluate the rate capacity, GCD profiles at various current densities in the range of 2.5–25 mA cm^−2^ were characterized, as shown in Fig. 5e. All the GCD profiles presented approximately symmetric shapes, demonstrating the excellent chemical reversibility during the GCD process, which was in good agreement with the CV results and confirmed the pseudocapacitive nature of the 3D Ni_3_S_2_ nanorod array electrode. The areal capacity was calculated for S-120-18, as summarized in Fig. 5f. It can be seen that the areal capacity decreased with increasing discharge current, which was assigned to the low utilization of the active materials and the increasing polarization at higher charge/discharge current. The areal capacity of S-120-18 decreased from 1370 µA h cm^−2^ to 877 µA h cm^−2^ (64% retention) in the current density ranges of 2.5 to 25 mA cm^−2^. Among the Ni_3_S_2_ nanorod electrodes, the excellent performances of S-120-18 can be attributed to its areal nanorod density to supply both enough surface for the electrochemical reaction and space for the transport of the electrolyte.

The capacitive performance of S-100-16, S-120-16 and S-140-16 was also investigated (Fig. S3–S5†), systematically, and they displayed similar phenomena to S-120-18. For S-120-16, when the current density increased from 5 to 25 mA cm^−2^, its areal capacity decreased from 1602 µA h cm^−2^ to 663 µA h cm^−2^ (Fig. S3c†).

**Fig. 6:**
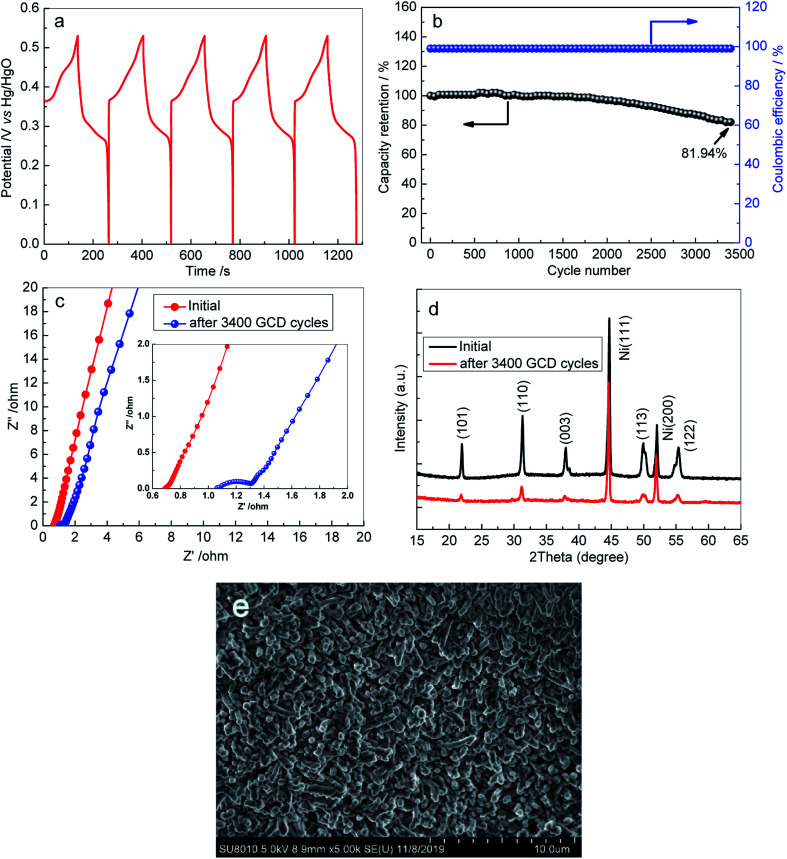
(a) The first five charge–discharge cycles of S-120-18 electrode; (b) cycling stability of S-120-18 electrodes at current densities of 15 mA cm^−2^; (c) Nyquist plots of S-120-18 electrodes before and after 3400 GCD cycles at 15 mA cm^−2^ over the frequency range of 0.01 to 10^5^ Hz. (d) XRD pattern of S-120-18 before and after the cycling tests, (e) SEM image of S-120-18 after the cycling tests.

Besides the areal capacity and rate capability, the long-term cycling stability of the 3D Ni_3_S_2_ nanorod arrays was evaluated by conducting a charge–discharge test at a constant current density of 15 mA cm^−2^. The charge–discharge voltage profiles of the first 5 cycles for S-120-18 are presented in Fig. 6a, which shows a very high coulombic efficiency. From the plot of capacity *versus* the cycle number curve (Fig. 6b), it can be seen that the capacity retention remained at 81.94% and the corresponding coulombic efficiency remained within the range of 99.5–100% after 3400 cycles, indicating the outstanding cycling behaviour of the 3D Ni_3_S_2_ nanorod array electrode. After a long-term cycling test, the *R*_s_ value became 1.09 Ω, which was only a small change from its initial value of 0.72 Ω (Fig. 6c). After the long-term cycling test, the 3D Ni_3_S_2_ nanorod electrode retained the stable crystal structure (Fig. 6d), and moreover, the SEM image showed that Ni_3_S_2_ still retains the nanorod nature after cycling tests (Fig. 6e). The results demonstrate the good stability of the capacitive performances of the 3D Ni_3_S_2_ nanorod array electrode.

The good areal capacity, rate capability and cycling behavior of the samples could be attributed to the porous three dimensional structure. Ni_3_S_2_ nanorod arrays were directly grown on Ni foam, which ensured the fine electrical contact and benefited the fast charge transfer between the interface of the Ni_3_S_2_ nanorod array electrode and Ni foam. In addition, the interconnected network with a porous structure facilitated transport of the electrolyte.

### 3.2. Catalytic properties of the 3D Ni_3_S_2_ nanorod arrays

In alkaline solutions (pH = 14), the overall water splitting is composed of the HER on the cathode and OER on the anode of the electrolyzer, which can be written as the following equation:^61^

Cathode:4H_2_O + 4e → 2H_2_ + 4OH^−^, *E*^0^(C) = +0.826 V

Anode:4OH^−^ → O_2_ + 2H_2_O + 4e, *E*^0^(A) = −0.404 VHere, *E*^0^(C) and *E*^0^(A) are the equilibrium half-cell potential under standard conditions of 1 atm and 25 °C. The overall water splitting process is thus2H_2_O → 2H_2_ + O_2_

**Fig. 7:**
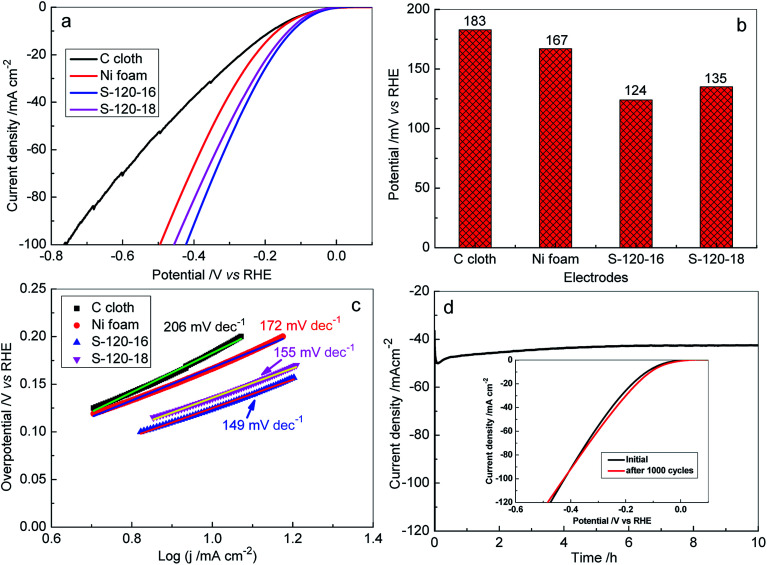
(a) LSV curves for the HER recorded on different electrodes: C cloth, Ni foam, S-120-16 and S-120-18 in 1.0 M KOH at a scan rate of 5 mV s^−1^; (b) bar plots obtained for the HER overpotential values *versus* the corresponding current density of 10 mA cm^−2^ for the different electrodes; (c) the corresponding Tafel plots for the HER on various electrodes; (d) time dependence of the current density of the S-120-16 electrode at a static overpotential of 122 mV. Inset: LSV curves of the S-120-16 electrode initially and after 1000 CV scans.

In the study, the HER catalytic activities on the 3D Ni_3_S_2_ nanorod array electrode were investigated in 1 M KOH electrolyte by linear sweep voltammetry (LSV) (Fig. 6, 7 and S6†). For comparison, the HER catalytic activities of 3D Ni_3_S_2_ nanorod arrays of S-120-16, S-120-18, Ni foam and C paper were investigated, systematically, as shown in Fig. 7. Interestingly, S-120-16 and S-120-18 can provide a geometric current density of 10 mA cm^−2^ at only 124 mV and 135 mV, respectively, which are much lower than 167 mV for Ni foam and 183 mV for C cloth (Fig. 7b). These performances are superior to those of many reported catalysts for the HER, including Ni_3_S_2_ nanosheets/NF (223 mV),^62^ Ni(OH)_2_/Ni_3_S_2_/NF (180 mV),^63^ Ni_2_P (150 mV),^63^ NiCoP nanotubes (150 mV),^64^ Ni–Sn@C(160 mV),^65^ Mo_2_C nanoparticles (190 mV),^66^ CoO_*x*_@CN (232 mV),^67^ CoO_*x*_@AC (270 mV),^67^ and so on (Fig. S7†).

Based on the LSV polarization curves, the corresponding Tafel plots were calculated. As shown in Fig. 7c, the Tafel slopes for S-120-16, S-120-18, Ni foam and C cloth are 149 eV dec^−1^, 155 eV dec^−1^, 172 eV dec^−1^, and 206 eV dec^−1^, respectively. Obviously, Tafel slopes on Ni_3_S_2_ nanorod array electrodes are much lower than those for the reaction on Ni foam and C cloth, indicating a rapid H_2_ generation reaction on the Ni_3_S_2_ nanostructural catalyst.^68^ The stability of the Ni_3_S_2_ nanorod array electrode was evaluated by amperometry (*i*–*t*) analysis. Fig. 7d shows the *i*–*t* curve at 122 mV for S-120-16, which presents a negligible degradation of current density for H_2_ generation for 10 h. In particular, after 1000 CV cycles tested at a scan rate of 20 mV s^−1^, the polarization curve is almost the same as the initial one, indicating no observable decay after long-term cycling (Fig. 7d inset). The results show the potential usage of the 3D Ni_3_S_2_ nanorod array catalyst in water splitting for a long time.

**Fig. 8:**
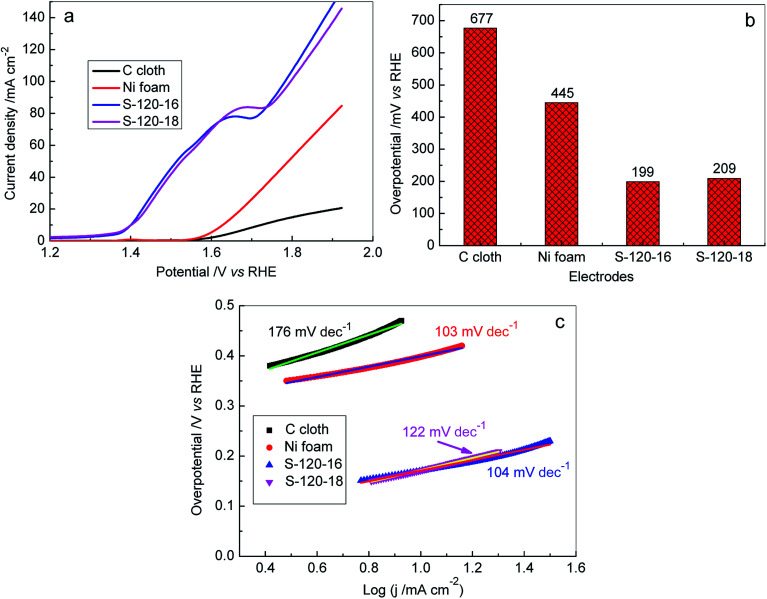
(a) LSV curves of the OER recorded on different electrodes: C cloth, Ni foam, S-120-16 and S-120-18 in 1.0 M KOH at a scan rate of 5 mV s^−1^; (b) bar plots obtained for the OER overpotential values *versus* the corresponding current density of 20 mA cm^−2^ for the different electrodes; (c) the corresponding Tafel plots for the OER on various electrodes.

On the other hand, the OER on the Ni_3_S_2_ nanorod array electrode was investigated in 1 M KOH solutions. The obtained results are illustrated in Fig. 8, S6c and d.† Obviously, the highly efficient OER catalytic activities were obtained for Ni_3_S_2_ nanorod array electrodes and their overpotentials were much lower than those of Ni foam and C cloth electrodes (Fig. 8a and b) at the same current density. At a current density of 20 mA cm^−2^, the overpotential dropped in the given sequence: C cloth (677 mV) < Ni foam (445 mV) < S-120-18 (209 mV) < S-120-16 (199 mV), as shown in Fig. 7b. We also calculated the overpotentials at 10 mA cm^−2^ for S-120-16 and S-120-18 electrodes and they were 170 mV and 171 mV, respectively, which are much lower than those of the other electrodes recently reported in the literature (Fig. S8†).^69–76^ Tafel plots were used to investigate the OER kinetics of Ni_3_S_2_ nanorod array electrodes, as shown in Fig. 8c. The Tafel slopes of S-120-16 and S-120-18 electrodes were calculated to be 104 mV dec^−1^ and 122 mV dec^−1^, respectively, which were lower than those of C and Ni foam. The results show that 3D Ni_3_S_2_ nanorod array electrodes are favourable for OER reaction kinetics.

**Fig. 9:**
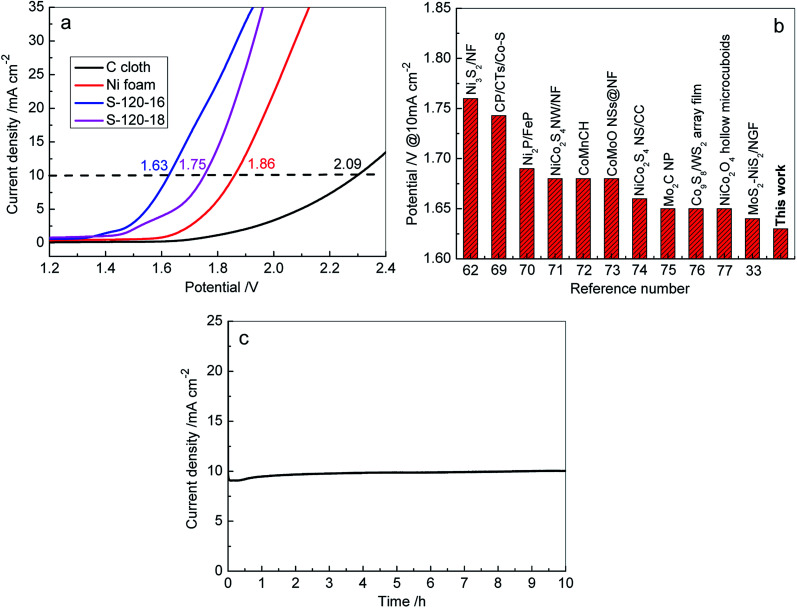
(a) LSV curves recorded for overall water splitting on C cloth, Ni foam, S-120-16 and S-120-18 electrodes in a two-electrode configuration at a scan rate of 5 mV s^−1^; (b) comparison of cell voltage for water electrolysis between Ni_3_S_2_ nanorod arrays (S-120-16) and the other recently reported electrolyzers at 10 mA cm^−2^; numbers are references cited; (c) time dependence of the current density of the S-120-16 electrode at a static potential of 1.63 V.

To further investigate the practical applicability of 3D Ni_3_S_2_ nanorod array electrodes as bifunctional electrodes for overall water splitting, the electrolyzer was assembled in 1 M KOH solution with a two-electrode using Ni_3_S_2_ nanorod arrays/NF as the anode and cathode. For comparison, the C cloth-based and Ni foam based electrolyzers were also assembled and tested under identical conditions, respectively. Fig. 9 shows the electrochemical performances of various electrolyzers in 1 M KOH solution. Both C cloth and Ni foam based electrolyzers demonstrated the very low activities for overall water splitting, exhibiting the cell voltages of 2.09 V and 1.86 V, respectively, at a current density of 10 mA cm^−2^. In contrast, the 3D Ni_3_S_2_ nanorod array electrode based electrolyzers achieved much lower cell voltages (1.63 V for S-120-16 and 1.75 V for S-120-18). In particular, the S-120-16 based electrolyzer shows a cell voltage of 1.63 V at a current density of 10 mA cm^−2^, which is significantly better than those of recently reported electrolyzers (Fig. 9b).^33,62,77–85^ Furthermore, we have also measured the long-term stability of 3D Ni_3_S_2_ nanorod array electrode (S-120-16) based electrolyzers, as shown in Fig. 9c. At a given potential of 1.63 V, the electrolyzer delivered an initial current density of 9.0 mA cm^−2^, and then the current density increased to ∼10.0 mA cm^−2^ in 40 min, after which, it displayed negligible change for water electrolysis in 10 h, indicating the outstanding stability of the 3D Ni_3_S_2_ nanorod array electrode. The excellent overall water splitting performances of 3D Ni_3_S_2_ nanorod arrays can be attributed to the large active surface area of the nanostructure which provided more active sites for electrochemical redox reactions.^86–88^ Besides, the porous three dimensional structure of the Ni_3_S_2_ nanorod array electrode offered rapid access for surface absorption and the transfer of OH^−^ ions in HER and OER processes for water splitting.^79^

## 4. Conclusions

In summary, controlled 3D Ni_3_S_2_ nanorod arrays were fabricated on Ni foam by a facile solvothermal route. The Ni_3_S_2_ nanorod arrays stood closely to each other on Ni foam. As a supercapacitor electrode, 3D Ni_3_S_2_ nanorod arrays exhibited outstanding performances. The areal capacity was as high as 1602 µA h cm^−2^ at 5 mA cm^−2^ and the capacity retention remained at 81.94% after 3400 charge–discharge cycles. In addition, when the Ni_3_S_2_ nanorod arrays were used as electrode materials for water splitting, they demonstrated superior electrocatalytic activity and excellent stability. The overpotentials for the HER and OER were 124 mV (at 10 mA cm^−2^) and 199 mV (at 20 mA cm^−2^), respectively, in 1.0 M KOH as the electrolyte. In particular, with the 3D Ni_3_S_2_ nanorod arrays as both the anode and cathode, the efficient electrolyzer for overall water splitting was assembled and it exhibited an outstanding capability to achieve 10 mA cm^−2^ with a cell voltage of 1.63 V and super durability for overall water splitting. The overall catalytic properties of 3D Ni_3_S_2_ nanorod arrays were attributed to the large active surface area of nanorods and their porous three dimensional structure. Therefore, the study presents a promising multifunctional 3D electrode material and opens new opportunities for electrochemical energy storage and conversion applications.

## Conflicts of interest

There are no conflicts to declare.

## Supplementary Material

NA-002-C9NA00633H-s001
