# Supplementary material for: Three dimensional Ni3S2 nanorod arrays as multifunctional electrodes for electrochemical energy storage and conversion applications
Source: Nanoscale Adv. 2019 Nov 22;2(1):478–88. doi: 10.1039/c9na00633h (PMC9417280; doi:10.1039/c9na00633h)
Supplement: NA-002-C9NA00633H-s001 [file NA-002-C9NA00633H-s001.pdf]

Electronic Supplementary Material (ESI) for Nanoscale Advances.
This journal is © The Royal Society of Chemistry 2019

Supporting Information

Three dimensional Ni_3S_2 nanorod arrays as multifunctional electrodes for electrochemical energy storage and conversion applications

Kexin Cui[a], Jincheng Fan[a,*], Songyang Li[a], Moukaila Fatiya Khadidja[a], Jianghong Wu[a,b], Mingyu Wang[a], Jianxin Lai[a], Hongguang Jin[a], Wenbin Luo[a], Zisheng Chao[a,*]

[a]*College of Materials Science and Engineering, Changsha University of Science and Technology, Changsha,Hunan 410114, China*

[b]*College of Health Science and Environmental Engineering, Shenzhen Technology University, Shenzhen, Guangdong 518118, China*

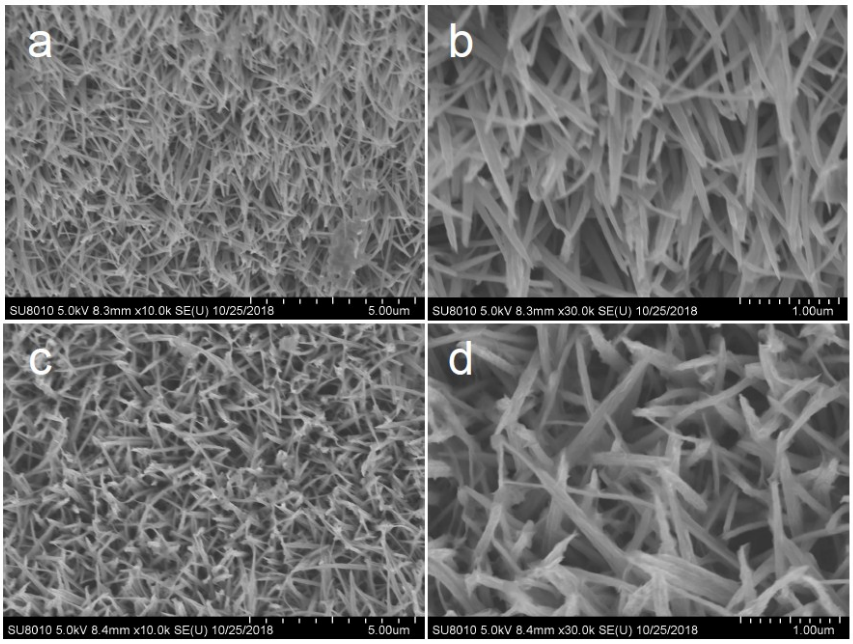

Figure S1. SEM images of the Ni_3S_2 nanorods on Ni foam with different magnifications for S-100-16 (a and b) and S-140-16 (c and d), (a) and (c) ×10k; (b) and (d) ×30k.

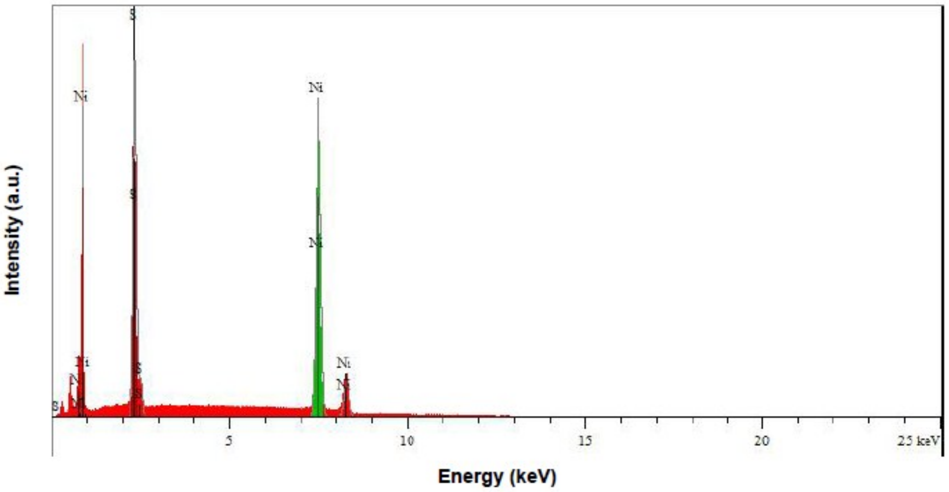

Figure S2. EDS elemental analysis of Ni_3S_2 nanorods on Ni foam. Only Ni and S peaks were observed, indicating the purity of the synthesized Ni_3S_2 nanorods.

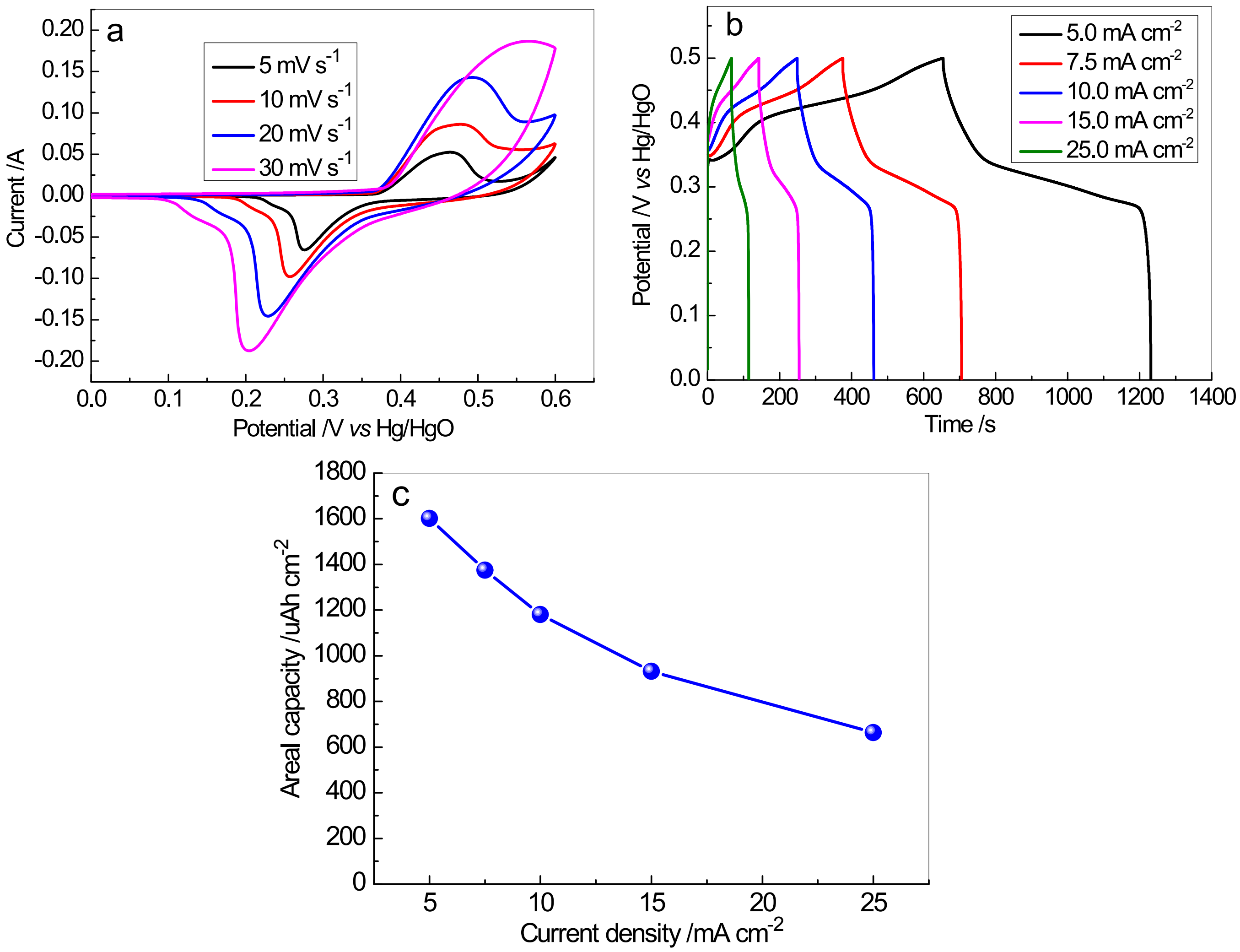

Figure S3. (a) CV curves obtained for S-120-16 electrode at different scan rates; (b) GCD profiles obtained for S-120-16 electrode at different current density; (c)The areal capacitance calculated from the discharge process at different current density.

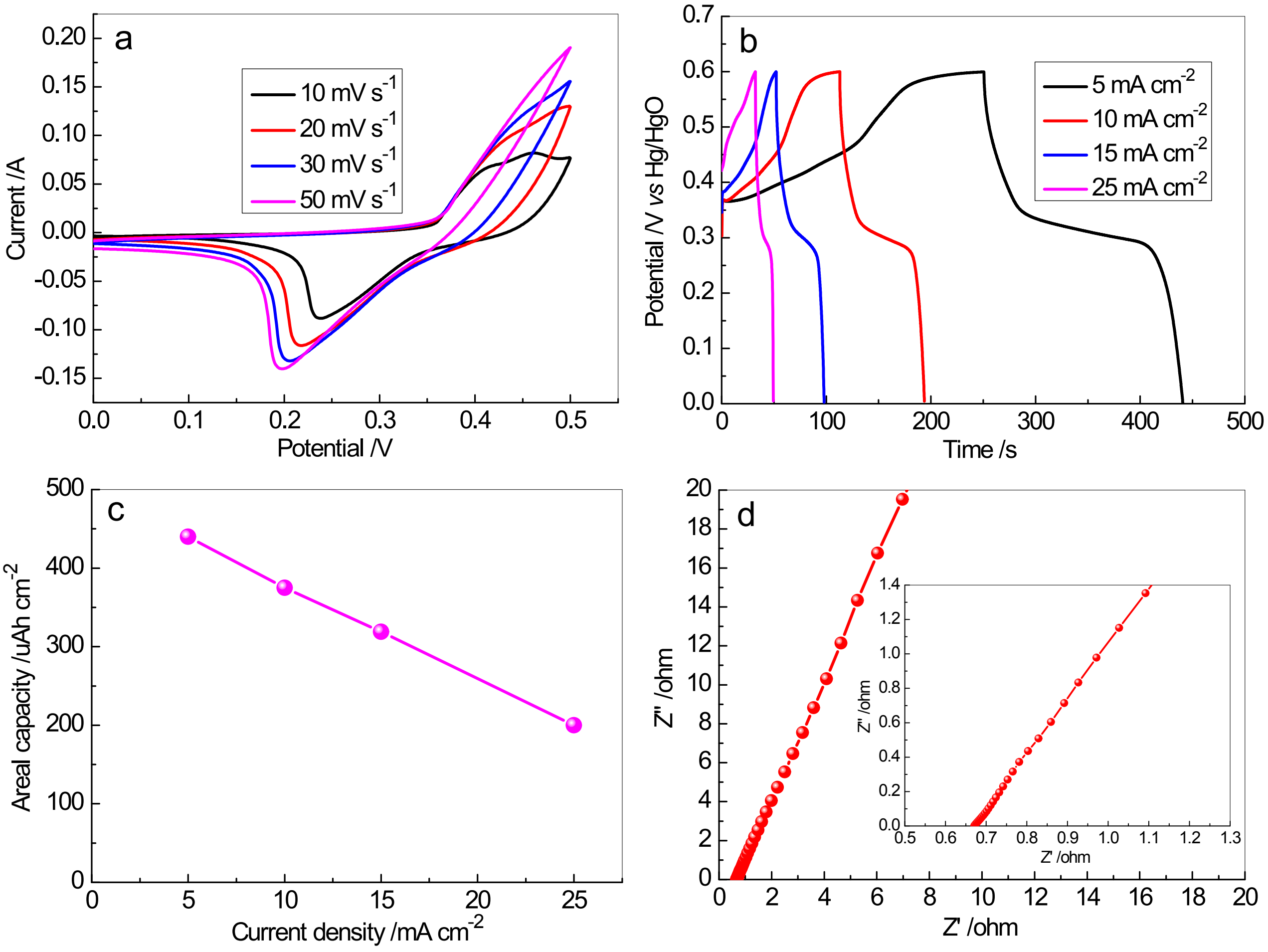

Figure S4. (a) CV curves obtained for S-140-16 electrode at different scan rates; (b) GCD profiles obtained for S-140-16 electrode at different current density; (c) The areal capacitance calculated from the discharge process at different current density; (d) Nyquist plots of S-140-16.

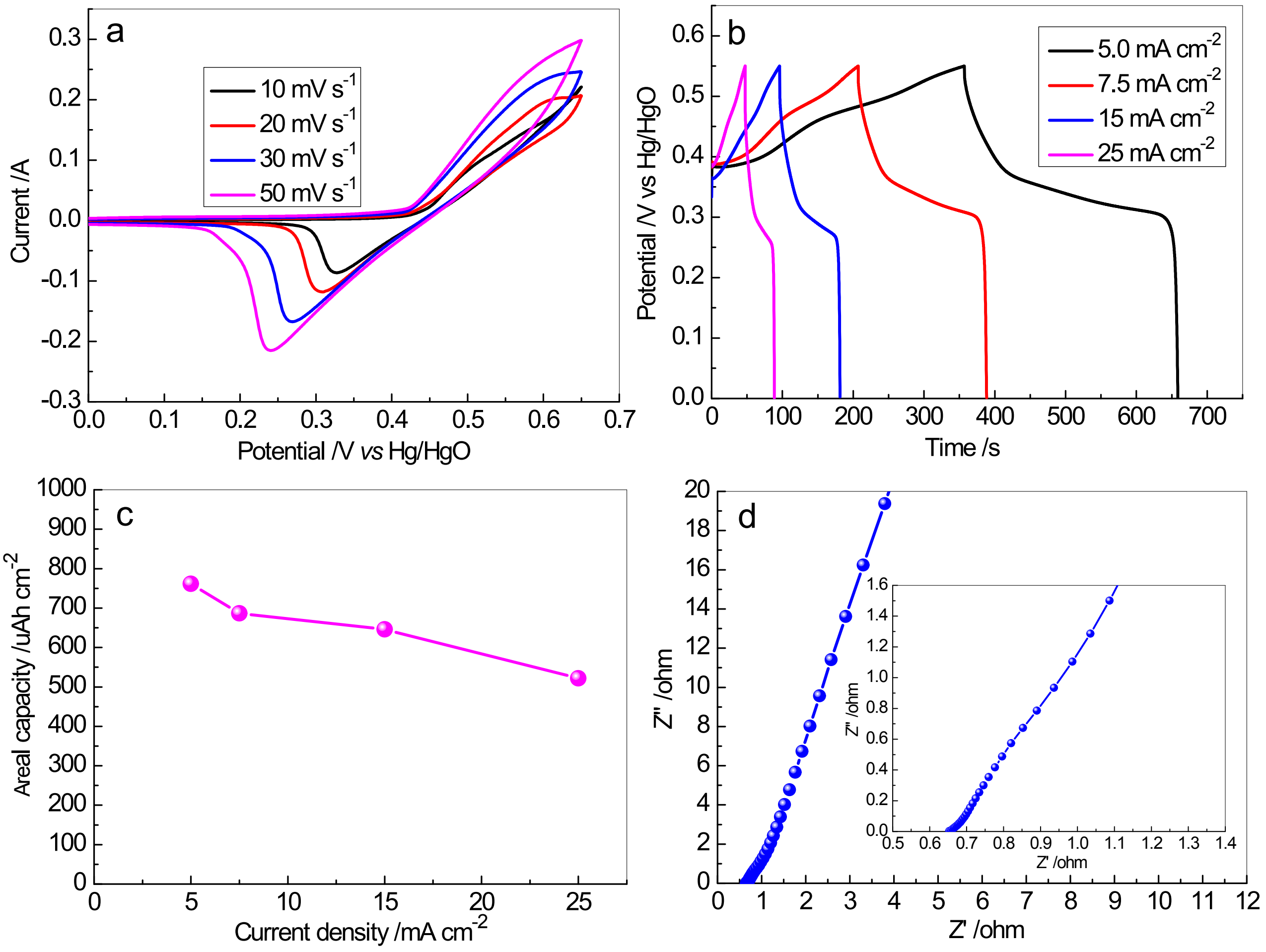

Figure S5. (a) CV curves obtained for S-100-16 electrode at different scan rates; (b) GCD profiles obtained for S-100-16 electrode at different current density; (c)The areal capacitance calculated from the discharge process at different current density; (d) Nyquist plots of S-100-16.

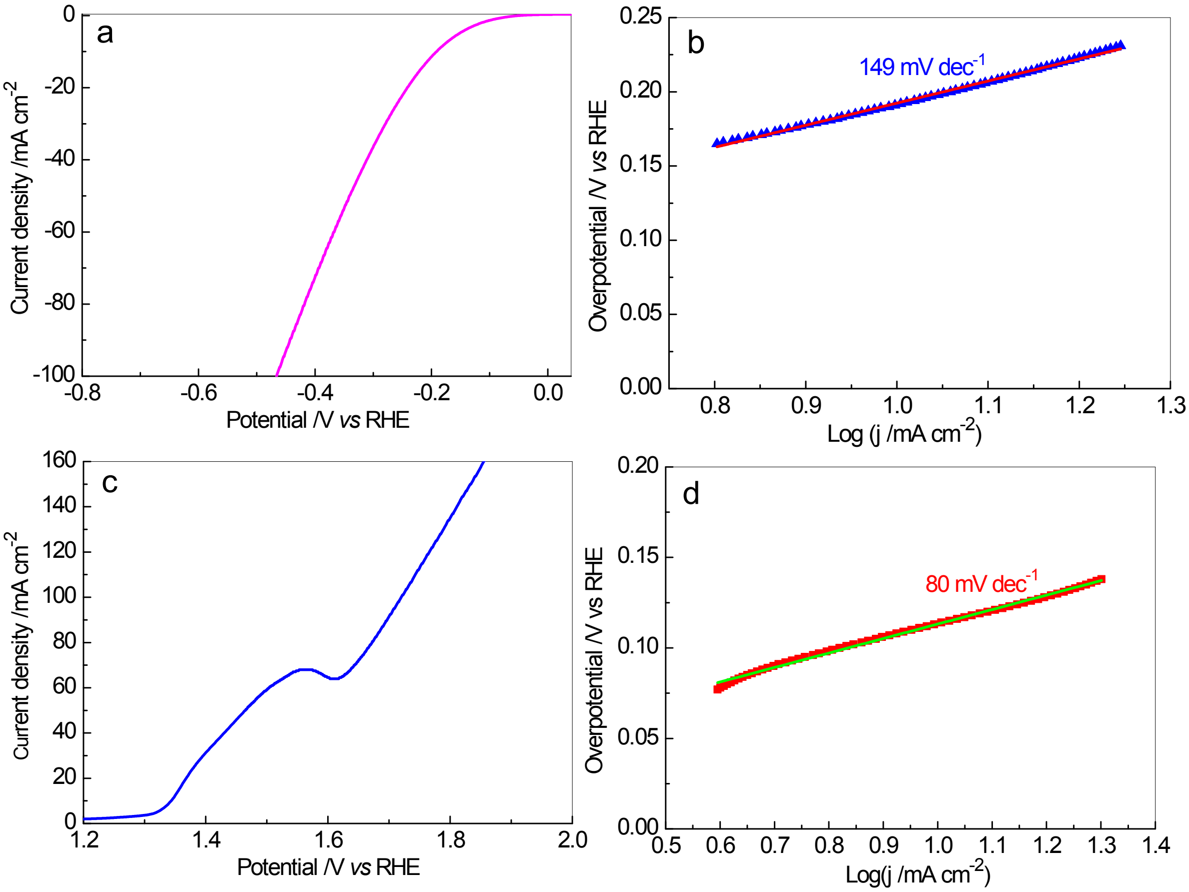

Figure S6. LSV curves recorded in 1.0 M KOH at a scan rate of 5 mV s^{-1} on S-140-16,(a) LSV curve for HER, (b)The corresponding Tafel plots for HER; (c) LSV curve for OER, (d)The corresponding Tafel plots for OER.

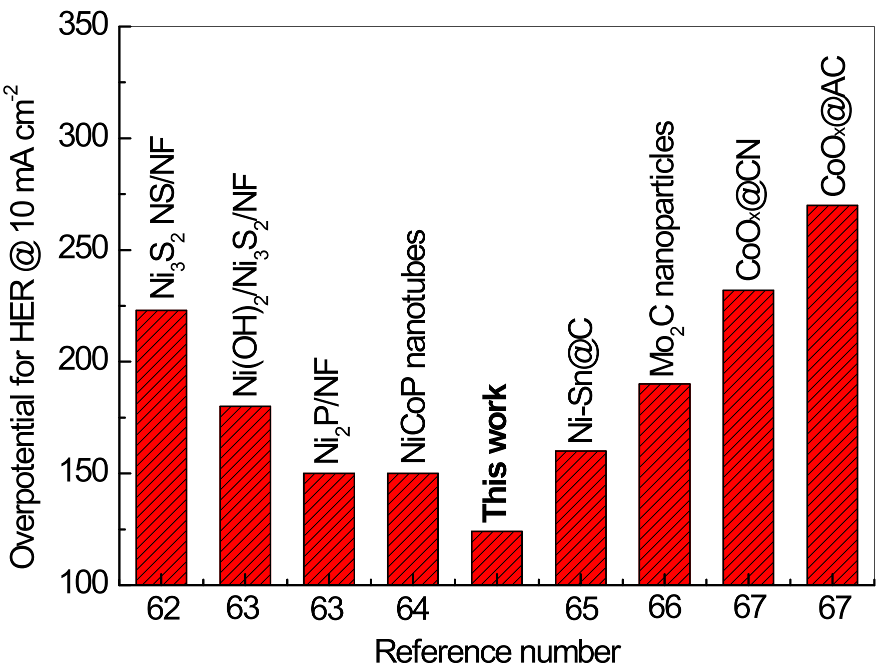

Figure S7. Comparison of overpotential values to achieve 10 mA cm^{-2} between Ni_3S_2 nanorod arrays (S-120-16) and the other recently reported HER catalysts , Numbers are references cited. The of overpotential Ni_3S_2 nanorod arrays was much lower than those of the other reported HER catalysts.

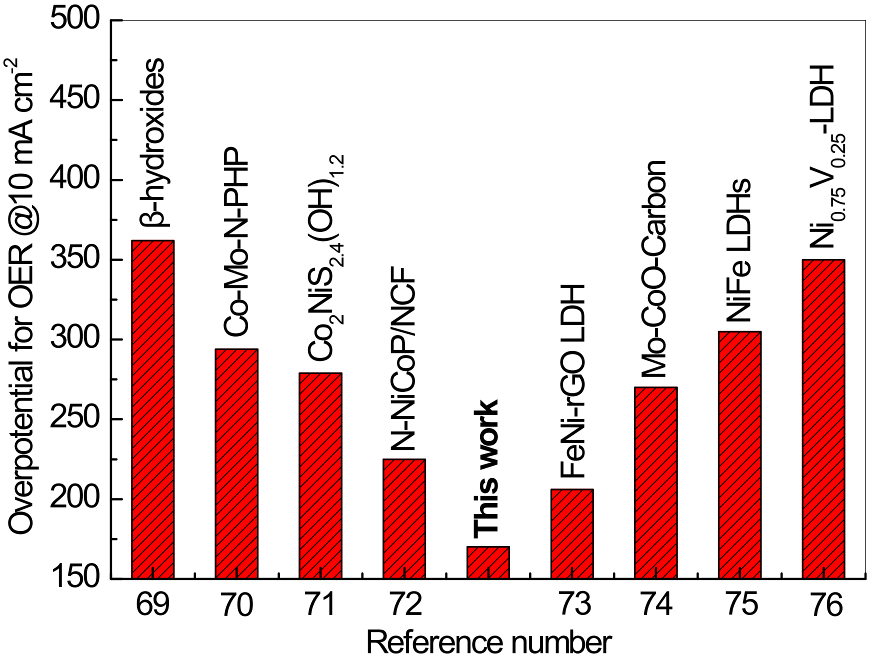

Figure S8. Comparison of overpotential values to achieve 10 mA cm^{-2} between Ni_3S_2 nanorod arrays (S-120-16) and the other recently reported OER catalysts , Numbers are references cited. The of overpotential Ni_3S_2 nanorod arrays was much lower than those of the other reported OER catalysts.
